# Re-Admission of COVID-19 Patients Hospitalized with Omicron Variant—A Retrospective Cohort Study

**DOI:** 10.3390/jcm11175202

**Published:** 2022-09-02

**Authors:** Irit Ayalon-Dangur, Adi Turjeman, Bar Basharim, Noa Bigman-Peer, Einat Magid, Hefziba Green, Tzippy Shochat, Alon Grossman, Jihad Bishara, Noa Eliakim-Raz

**Affiliations:** 1 Internal Medicine E, Rabin Medical Center, Beilinson Hospital, Petah Tikva 49100, Israel; 2 Sackler Faculty of Medicine, Tel Aviv University, Tel Aviv 69978, Israel; 3 Research Authority, Rabin Medical Center, Beilinson Hospital, Petah-Tikva 49100, Israel; 4 Internal Medicine A, Kaplan Medical Center, Rehovot 76100, Israel; 5 Faculty of Medicine, Hebrew University of Jerusalem, Jerusalem 9112001, Israel; 6 Bio-Statistical Unit, Rabin Medical Center, Beilinson Hospital, Petah Tikva 49100, Israel; 7 Internal Medicine B, Rabin Medical Center, Beilinson Hospital, Petah Tikva 49100, Israel; 8 Infectious Diseases Unit, Rabin Medical Center, Beilinson Hospital, Petah-Tikva 49100, Israel

**Keywords:** COVID-19, SARS-CoV-2, Omicron variant, re-admissions

## Abstract

In accordance with previous publications, re-admission rates following hospitalization of patients with COVID-19 is 10%. The aim of the current study was to describe the rates and risk factors of hospital re-admissions two months following discharge from hospitalization during the fifth wave due to the dominant Omicron variant. A retrospective cohort study was performed in Rabin Medical Center, Israel, from November 2021 to February 2022. The primary outcome was re-admissions with any diagnosis; the secondary outcome was mortality within two months of discharge. Overall, 660 patients were hospitalized with a diagnosis of COVID-19. Of the 528 patients discharged from a primary hospitalization, 150 (28%) were re-admitted. A total of 164 patients (25%) died throughout the follow-up period. A multi-variable analysis determined that elevated creatinine was associated with a higher risk of re-admissions. Rates of re-admissions after discharge during the Omicron wave were considerably higher compared to previous waves. A discharge plan for surveillance and treatment following hospitalization is of great importance in the management of pandemics.

## 1. Introduction

Since the beginning of the SARS-Co-V-2 pandemic, hospitalization worldwide created an enormous clinical and economic burden [1]. Reported re-admission rates during the first COVID-19 waves were 8.97%, with most hospitalizations and mortalities occurring within a month following discharge [2]. Previous studies have identified parameters and risk factors that can predict re-admission rates [3,4]. The emergence of the fifth wave in Israel, the Omicron wave, commenced on 26 November 2021. Subsequently, the Omicron variant has spread rapidly throughout the country. The fifth wave was characterized by slightly different clinical features compared to previous waves. Recent publications have indicated a lower risk of hospitalization and mortality seen with the Omicron variant [5,6,7]. We therefore question as to whether the long-term outcome, including mortality and repeated hospitalizations, will also elicit different results.

The aim of the current study was to describe the rates and risk factors for re-admissions with any diagnosis two months following discharge from COVID-19 hospitalization during the fifth wave with the dominant Omicron variant.

## 2. Materials and Methods

### 2.1. Study Design

A retrospective cohort study was performed in Rabin Medical Center, a tertiary center, comprising Beilinson and Hasharon Hospitals, from November 2021 to February 2022. The study was approved by the Rabin Medical Center’s (RMC) Institutional Review Board with a waiver of written informed consent.

### 2.2. Study Population

All patients >18 years of age who were hospitalized in the COVID-19 wards, internal medicine wards or geriatric wards with a confirmed SARS-CoV-2 diagnosis, regardless of the degree of severity and duration of hospitalization, were included. Data were collected on re-admissions for a second hospitalization to any department in any Israeli hospital, for any diagnosis, regardless of duration of hospitalization. Exclusion criteria included patients younger than 18 years of age and pregnant women.

### 2.3. Data Collection, Surveillance and Follow-Up

Baseline and demographic characteristics, comorbidities according to the Charlson comorbidity index (CCI) [8], solid organ transplantation, functional and cognitive status and data relating to COVID-19 hospitalization, i.e., vital signs on admission, relevant laboratory values, severity of COVID-19 disease, medical therapy, oxygen support, length of stay (LOS) and discharge placement, were collected and extracted from the computerized electronic system of RMC. Re-admission information was also collected and included vital signs, relevant laboratory tests, time to re-admissions, re-admissions diagnoses and LOS. A diagnosis of COVID-19 was confirmed by a positive respiratory polymerase chain reaction (PCR) test for SARS-CoV-2. The severity of the COVID-19 disease was defined according to the National Institute of Health’s (NIH) guidelines, adopted by the Israel Ministry of Health [9]. Medical therapy for COVID-19 during hospitalization was based on the NIH and WHO guidelines [9,10], on local guidelines and on recent literature.

### 2.4. Outcome Assessments

The primary outcome was re-admissions for any diagnosis 60 days following discharge from the primary COVID-19 hospitalization. The secondary outcome was all-cause mortality throughout the follow-up period. Date of death was retrieved through the hospital administration’s data system, which is constantly updated in conjunction with the Israeli’s Ministry of Interior’s data, and included out of hospital or in-hospital mortality. Re-admissions were extracted from the patients’ medical computerized system records.

### 2.5. Statistical Analysis

The statistical analysis was generated using SAS Software, Version 9.4. Continuous variables were presented by mean ± standard deviation, and categorical variables were presented by (N, %). The Cox Proportional Hazard Model, with the Fine and Gray correction for Death as a competing risk, analyzed the primary endpoint of re-admission and all univariate and multivariate analyses. The Cox Proportional Hazard Model analyzed the secondary endpoint of death. Two-sided *p* values less than 0.05 were considered statistically significant.

## 3. Results

### 3.1. Study Population

Overall, 660 patients were hospitalized with a diagnosis of COVID-19 and were included in the final analysis. Of the 528 patients discharged from the primary hospitalization, 150 (28%) were re-admitted during the two-month follow-up period. The baseline characteristics of re-admitted patients compared with the non-re-admitted patients are presented in Table 1. Age, gender and BMI were equally distributed between the two groups. The mean age of the total cohort was 73.9 ± 15.5 years. A total of 357 patients (54%) were male. The mean CCI score of the total cohort was 5.5 ± 3.4.

### 3.2. Mortality throughout the Follow-Up Period

Throughout the follow-up period, 164 patients (25%) died; of them, 132 (20%) died during the primary COVID-19 hospitalization. Twenty patients (3%) were re-admitted and subsequently died; 12 (2%) died after the COVID-19 hospitalization but were not re-admitted.

### 3.3. Risk Factors for Re-Admission

Re-admission rates were higher in patients > 60 years of age compared to the younger patients (134 patients (89%) vs. 275 patients (75%), in the re-admitted patients compared to the non-re-admitted patients respectively, *p* = 0.01) (Table 1). CCI was significantly higher in the re-admitted patients. Renal disease and kidney transplantation and heart and cardio-vascular diseases were more prevalent in the re-admitted group. The severities of COVID-19 infection were not associated with re-admission, except for patients with critical COVID-19, who tended to be re-admitted more frequently. Treatments during the primary hospitalization that included the new agents Paxlovid and Molnupiravir were not associated with re-admission. The median D-dimer level was significantly higher in the re-admitted group compared to the non-re-admitted group (1438 (204–6093) and 1174 (124–40,975) ng/mL respectively, *p* = 0.03). Serum creatinine levels were also significantly higher in the re-admitted group (2 vs. 1.4 mg/dL, respectively, *p* = 0.01). The mean duration of the primary COVID-19 hospitalization was 7 days in both groups and was not found to be a risk factor for re-admissions.

In multivariable analysis, which included age, gender, BMI, CCI score, department of hospitalization before current hospitalization and serum creatinine (Table 2), increased serum creatinine was found to be the only independent and significant risk factor for re-admission (HR 1.37, 95% CI 1.05 to 1.78, *p* = 0.02).

### 3.4. Clinical Characteristics of Re-Admitted Patients

Causes for re-admissions are described in Table 3. In total, 48 patients (32%) presented with an active COVID-19 infection at the time of re-admission, and 94 (63%), when re-admitted, were classified as recovered. The COVID-19 status of the rest was unknown. The mean time from discharge to re-admission was 13 ± 13 days, and the mean duration of re-admission was 8 ± 6 days. Twelve patients were re-admitted 24 hours after discharge; of them, 9 (75%) presented with a diagnosis of active COVID-19. The most common diagnoses on re-admission included infectious causes (pneumonia, skin and soft tissue infection, urinary tract infection) and diseases of the gastrointestinal (GI) tract (GI bleeding, cholelithiasis, cholangitis) and cardio-vascular system (congestive heart failure exacerbation and chest pain). The mean level of vital signs on re-admission were within the normal range. Creatinine on re-admission was higher than normal (mean 2.0 ± 1.8 mg/dL).

## 4. Discussion

In this retrospective cohort study of patients hospitalized during the Omicron variant’s fifth wave of the pandemic, re-admission rates were 28%, which is considerably higher than was previously demonstrated for the other variants. A previous Israeli study conducted during the fourth wave found that in a median post-discharge follow-up of 59 days, 9.2% of the patients were re-admitted [3]. This is compatible with a systematic review and meta-analysis published in January 2022 [2], which found re-admission rates of 8.97% 30 days post-discharge. In view of the fact that for many of our patients the follow-up period was relatively short, these numbers may be an underestimation of the actual re-admission rate. A possible explanation for the higher rate of re-admissions in our study is that the population who required hospitalization due to the Omicron variant had significant comorbidities, with a very high mean CCI score of 5.5 (higher compared to the 2.4 observed in a previous Israeli study during the fourth wave [3]). Notably, hospitalization duration was longer in the current study with the Omicron variant compared to a previous Israeli study with the Delta variant [3]. This can probably be attributed to the significant comorbidities of our study population. The re-admission rate in our cohort was also considerably higher compared to the literature following hospitalization in an internal medicine ward in non-COVID-19 patients. In one Israeli study, the rate of re-admissions within 30 days of discharge was 12.2% [11]. Other studies have observed between 5.2% and 17.5% re-admission rates [12,13] in non-COVID-19 patients. The rate of a 30-day re-admission after hospitalization with non-COVID-19 respiratory infections was 7.4% for influenza-positive patients and 16.7% for influenza-negative patients [14]. A re-admission rate of older adults with community-acquired pneumonia was 11.4% within 30 days of discharge [15] and 11.2% within 90 days of discharge [16]. Another explanation for the higher rate of re-admissions in the current study might be that the Omicron variant causes a mild upper respiratory tract infection; however, complications are delayed for a few days following a diagnosis with a biphasic clinical pattern. Mortality rates were relatively high (25%) in our cohort. A recently published multi-center study from Israel comparing the COVID-19 pandemic waves in hospitalized patients found that the 30-day mortality was 14% during the first and second waves and 25% during the third wave. The mortality rates in our cohort were similar to mortality rates during the third wave [17]. The high incidence of mortality in our cohort may be attributed to the significant comorbidities of the study population. Another possible explanation may have been less dominant support systems in the fifth wave, such as institutions adjusted for the treatment of patients with COVID-19 following discharge. Availability and accessibility of such institutions, to be opened and closed with short notice, is of great importance to assist in the regulation of hospitalizations. 

The importance of interventions with a post-discharge follow-up was demonstrated in previous studies and was shown to reduce re-admission rates, especially for the older population [18,19]. Finally, the psychological influence of COVID-19 on the medical staff has been demonstrated in previous studies [20,21], and perhaps, after five waves, their fatigue has begun to impact their performance. Higher serum creatinine was associated with a nearly 1.5 times increased risk for re-admission. Unexpectedly, COVID-19 severity did not influence re-admission rates, except for critically ill COVID-19 patients, who tended to be re-admitted more frequently. This finding may be explained by the assumption that repeated hospitalizations during the fifth wave were attributed to the patient’s comorbidities and not to the respiratory burden of COVID-19. Surprisingly, age was not found to be a risk factor for re-admission, as has been demonstrated in previous studies [3,22,23]; however, when stratifying according to age < 60 years and age ≥ 60 years, which is approximately the cutoff for severe COVID-19, a significantly higher rate of re-admissions was observed in the older population. Placement before current hospitalization, baseline functional capacity, LOS, vital signs on admission, and most laboratory parameters were also equally distributed between the two groups. In this study, unexpectedly, CRP levels were found to not be associated with the risk of re-admissions. This finding is in contrast to previous studies, which determined elevated CRP as a prognostic factor [24,25], and is opposed to a previous Israeli study [3] examining risk factors for re-admission during the fourth wave, in which CRP levels were found to be a risk factor. An explanation for this finding is that perhaps the inflammatory state of the COVID-19 disease was milder during the fifth wave and that morbidity due to the Omicron variant may be attributed to other mechanisms. Interestingly, treatment during hospitalization did not influence the risk for re-admission, including the new agents Paxlovid and Molnupiravir, despite the fact that treatment with these agents was associated with a lower risk of progression to severe COVID-19, a lower incidence of COVID-19-related hospitalization or death and a lower viral load in non-hospitalized patients [26,27]. The relatively small number of patients treated with these agents (30 and 36 patients with Paxlovid and Molnupiravir, respectively) and the fact that these patients were already hospitalized during treatment questions the strength of this finding. Furthermore, only 32% of re-admission diagnoses included an active COVID-19 disease, such that perhaps the treatment indeed reduced re-admissions of patients with active COVID-19. It is interesting to note that the rate of re-admission of patients with active COVID-19 disease was 32% in our study, much lower when compared to a previous Israeli study during a previous wave, where re-admission rates with active COVID-19 disease was 66% [3]. It is essential to mention that, in our study, it cannot be ruled out that the other 68% who were defined as recovered were not suffering from long-COVID or COVID-19 complications such as GI bleeding or thromboembolic events, in particular given that the mean time to re-admission was 13 days. Lastly, as mean time to re-admission was 13 days, this might influence other findings to identify risk factors for re-admission.

This study has several limitations. The first is that our study included only two medical centers; nonetheless, data were included regarding re-admissions from hospitals throughout Israel.

A second limitation is the fact that gene sequencing was not performed routinely and the confirmation of Omicrom variant in all patients was not undertaken; however, we assume that during this time period, the vast majority of patients were infected with the Omicron variant as indicated in formal reports of the Israel Ministry of Health and that only a minority were infected with the Delta variant.

A third limitation was the lack of data regarding the patients’ immunization status and the number of vaccinations each patient received. Yet, we assume that the majority of patients were vaccinated in view of the underlying comorbidities of the patient population in our cohort and in light of the great compliance of this population and the high vaccination rates in Israel. Another limitation stems from the retrospective nature of the study; however, since this study did not include a comparison between different interventions, the potential for selection bias was not significant.

## 5. Conclusions

We found that the rate of re-admissions after discharge from a COVID-19 hospitalization in Israel, during the fifth wave with the dominant Omicron variant, was considerably higher compared to the rates of re-admission during previous waves or after hospitalization in internal medicine wards with other respiratory illnesses. Moreover, the mortality rate in our cohort was notably high.

Evaluating re-admission and mortality rates and identifying risk factors for re-admissions will greatly assist in developing a discharge plan for surveillance, treatment and pandemic management, thereby reducing re-admissions, decreasing the burdens on the medical services and providing better care to patients.

## Figures and Tables

**Table 1:** Baseline characteristics of the study cohort: re-admitted patients compared with those who were not re-admitted.

	Alive, No Re-Admission (*n* = 366)	Readmission (*n* = 150)	Dead, No Readmission (* *n* = 144)	Total (*n* = 660)	*p* Value
Age (years), mean (SD)	70.6 (17.0)	75.0 (12.7)	81.3 (10.8)	73.9 (15.5)	0.18
Age < 60, (*n*, %)	91 (25)	16 (11)	4 (3)	111 (17)	0.01
Age ≥ 60, (*n*, %)	275 (75)	134 (89)	140 (97)	549 (83)	
Male gender, (*n*, %)	192 (52)	91 (61)	74 (51)	357 (54)	0.10
BMI, mean (SD)	*n* = 253 27.0 (5.5)	*n* = 115 26.1 (6.0)	*n* = 98 25.7 (4.6)	*n* = 466 26.5 (5.5)	0.44
Smokers, *n* (%)	27 (7)	22 (15)	15 (10)	64 (10)	0.07
Baseline functional capacity, (*n*, %)					0.68
Independent	185 (51)	63 (42)	30 (21)	278 (42)	
Needs assistance in ADL	172 (47)	85 (57)	110 (76)	367 (56)	
Cognitive status on admission, (*n*, %)					0.02
Oriented	281 (77)	119 (79)	62 (43)	462 (70)	
Not oriented	76 (21)	31 (21)	79 (55)	186 (28)	
Placement before current hospitalization					0.55
Home	253 (69)	104 (69)	94 (65)	451 (68)	
Medical institution	30 (8)	12 (8)	24 (17)	66 (10)	
Another hospital	22 (6)	7 (5)	7 (5)	36 (5)	
Vital signs on admission					
Saturation, median (IQR)	*n* = 255 96 (65–100)	*n* = 113 95 (80–100)	*n* = 106 94 (64–100)	*n* = 476 95 (64–100)	0.46
Fever, median (IQR)	*n* = 290 36.9 (35.6–39.5)	*n* = 124 36.9 (35.9–39.5)	*n* = 117 37.1 (33.8–39.0)	*n* = 531 36.9 (33.8–39.5)	0.81
Systolic blood pressure, mean (SD)	*n* = 302 137.5 (27.0)	*n* = 125 138.1 (29.2)	*n* = 122 127.7 (27.7)	*n* = 549 135.5 (27.8)	0.21
Diastolic blood pressure, mean (SD)	*n* = 302 71.8 (14.0)	*n* = 125 70.3 (14.3)	*n* = 122 67.0 (14.7)	*n* = 549 70.4 (14.3)	0.97
Heart, mean (SD)	*n* = 293 84.1 (17.3)	*n* = 124 85.4 (14.6)	*n* = 115 89.9 (18.4)	*n* = 532 85.6 (17.1)	0.97
Laboratory tests on admission, mean (SD)					
White blood cells (K/micl)	*n* = 357 9.1 (11.0)	*n* = 145 10.9 (13.4)	*n* = 143 12.4 (10.4)	*n* = 645 10.3 (11.5)	0.19
Lymphocytes (K/micl)	*n* = 357 2.1 (9.4)	*n* = 145 1.5 (6.0)	*n* = 143 2.3 (8.0)	*n* = 645 2.0 (8.5)	0.72
Hemoglobin (g/dL)	*n* = 357 12.4 (2.3)	*n* = 145 11.3 (2.6)	*n* = 143 11.8 (2.3)	*n* = 645 12.0 (2.4)	<0.0001
Platelates (K/micl)	*n* = 357 237.7 (123.6)	*n* = 145 231.2 (117.6)	*n* = 143 240.1 (119.9)	*n* = 645 236.8 (121.3)	0.58
C-reactive protein (mg/dL)	*n* = 351 7.7 (8.9)	*n* = 142 8.7 (7.5)	*n* = 141 13.7 (10.3)	*n* = 634 9.3 (9.2)	0.49
D-dimer (ng/ml), median (IQR)	*n* = 136 1174 (124–40975)	*n* = 50 1438 (204–6093)	*n* = 52 2172 (306–26812)	*n* = 238 1355 (124–40975)	0.03
Creatinine (mg/dL)	*n* = 360 1.4 (1.7)	*n* = 146 2.0 (1.8)	*n* = 141 2.0 (1.4)	*n* = 647 1.7 (1.7)	0.01
Bilirubin (mg/dL)	*n* = 354 0.6 (0.5)	*n* = 145 0.6 (0.7)	*n* = 139 0.7 (1.2)	*n* = 638 0.6 (0.7)	0.52
Ferritin (mg/ml), median (IQR)	*n* = 60 247.6 )8.3–1743.0)	*n* = 22 265.6 (39.0–1491.6)	*n* = 17 444.8 (118.1–4067.0)	*n* = 99 267.6 (8.3–4067.0)	0.67
Sodium (mg/dL)	*n* = 360 136.3 (5.1)	*n* = 146 136.7 (6.6)	*n* = 142 139.2 (10.9)	*n* = 648 137.0 (7.2)	0.61
Potassium (mg/dL)	*n* = 359 4.3 (0.8)	*n* = 146 4.5 (0.9)	*n* = 142 4.5 (0.9)	*n* = 647 4.4 (0.8)	0.18
Troponin (ng/l), median (IQR)	*n* = 124 39 (13–7027)	*n* = 54 47 (17–564)	*n* = 56 78 (16–3033)	*n* = 234 47 (13–7027)	0.20
Lactate (mg/dL), median (IQR)	*n* = 326 17 (4–126)	*n* = 136 15 (6–81)	*n* = 140 23 (6–114)	*n* = 602 17 (4–126)	0.04
Comorbidities, (*n*, %)					
Ischemic heart disease	70 (19)	52 (35)	33 (23)	155 (23)	0.0002
Congestive heart failure	51 (14)	47 (31)	29 (20)	127 (19)	<0.0001
Peripheral vascular disease	51 (14)	47 (31)	29 (20)	127 (19)	0.004
Diabetes melitus	105 (29)	58 (39)	54 (38)	217 (33)	0.10
Leukemia and multiple myeloma	16 (4)	4 (3)	7 (5)	27 (4)	<0.0001
Lymphoma	11 (3)	5 (3)	1 (0.7)	17 (3)	0.37
Cerebrovascular disease	74 (20)	36 (24)	33 (23)	143 (22)	0.40
Metastatic solid tumor	56 (15)	30 (20)	29 (20)	115 (17)	0.29
Dementia	4 (1)	1 (1)	4 (3)	9 (1)	0.45
Chronic pulmonary disease	57 (16)	30 (20)	28 (19)	115 (17)	0.40
Connective tissue disease	8 (2)	5 (3)	3 (2)	16 (2)	0.48
Peptic ulcer disease	27 (7)	15 (10)	11 (8)	53 (8)	0.21
Acquired Immune Deficiency Syndrome (AIDS)	1 (0.3)			1 (0.2)	<0.0001
Moderate to severe renal disease	13 (4)	13 (9)	3 (2)	29 (4)	0.01
Moderate to severe liver disease	19 (5)	4 (3)	3 (2)	26 (4)	0.32
Chronic obstructive pulmonary disease	17 (5)	9 (6)	4 (3)	30 (5)	0.39
Hypertension	130 (35)	86 (57)	65 (45)	281 (43)	<0.0001
Dyslipidemia	21 (6)	18 (12)	20 (14)	59 (9)	0.13
Charlson comorbidity index (CCI), mean (SD)	5.0 (3.6)	6.3 (3.1)	6.2 (2.8)	5.5 (3.4)	0.0003
Solid organ transplantation, (*n*, %)					
Kidney	7 (2)	10 (7)	5 (3)	22 (3)	0.01
Heart					
Liver					
Lung	3 (0.8)		1 (0.7)	4 (0.6)	<0.0001
Covid severity, (*n*, %)					0.06
Mild	169 (46)	66 (44)	8 (6)	243 (37)	
Moderate	49 (14)	19 (13)	4 (3)	72 (11)	
Severe	127 (34)	48 (32)	44 (31)	219 (33)	
Critical	21 (6)	17 (11)	88 (61)	126 (19)	
COVID-19 treatment, (*n*, %)					
Dexamethasone	127 (35)	48 (32)	44 (31)	219 (33)	0.33
Hydrocortisone	18 (5)	9 (6)	34 (24)	61 (9)	0.16
Prednisone	36 (10)	21 (14)	6 (4)	63 (10)	0.03
Clexane	197 (54)	89 (59)	109 (76)	395 (60)	0.74
Plasma	19 (5)	7 (5)	10 (7)	36 (5)	0.94
Tocilizumab	5 (1)		1 (0.7)	6 (0.9)	<0.0001
Baricitinib	24 (7)	7 (5)	18 (13)	49 (7)	0.21
Paxlovid	21 (6)	7 (5)	2 (1)	30 (5)	0.92
Molnupiravir	22 (6)	9 (6)	5 (3)	36 (5)	0.87
Ventilation during hospitalization	30 (8)	13 (9)	57 (60)	100 (15)	0.02
Discharge placement, (*n*, %)					
Home	363 (99)	149 (99)	20 (14)	532 (81)	
Another hospital	1 (0.3)			1 (0.2)	
House hospitalization	1 (0.3)			1 (0.2)	
Death during hospitalization			124 (86)	124 (19)	
Duration of hospitalization, mean (SD)	6.8 (7.0)	7.2 (7.0)	9.0 (7.1)	7.4 (7.1)	0.99

BMI = Body mass index; CCI = Charlson comorbidity index; SD = Standard deviation; ADL= Activities of daily living; IQR= Interquartile range. * 144 patients = 132 patients who died during their original hospitalization + 12 patients who died without re-admission

**Table 2:** Risk factors for re-admission according to a multi-variable analysis.

Parameter		HR (95% CI)	*p* Value
Age		1.00 (0.99–1.02)	0.85
Gender		1.30 (0.84–2.02)	0.23
BMI		1.02 (0.97–1.06)	0.45
CCI		1.05 (0.99–1.11)	0.10
Placement before current hospitalization	Medical institution (compared to home)	1.09 (0.56–2.13)	0.81
	Another hospital (compared to home)	1.04 (0.41–2.65)	0.94
Serum creatinine		1.37 (1.05–1.78)	0.02

HR = Hazard ratio; CI = Confidence interval; BMI = Body mass index; CCI = Charlson comorbidity index. For continuous variables HR given per 1 unit increment: year (for age), kg/m^2^ (for BMI), mg/dL (for creatinine).

**Table 3:** Clinical diagnoses of re-admission after hospitalization with COVID-19.

* Re-Admission Diagnoses, *n* = 150		*n* (%)
Infectious	Total	70 (47)
	Active COVID-19	48
	Pneumonia	11
	Skin and soft tissue infection	4
	Urinary tract infection	4
	GI infection	1
	Sepsis	2
General deterioration		6 (4)
Syncope		2 (1)
Respiratory	Total	8 (5)
	Dyspnea	6
	COPD exacerbation	2
Gastro-intestinal	Total	13 (9)
	Ascites	1
	Colonoscopy	1
	GI bleeding	4
	Cholelithiasis	2
	Cholangitis	2
	Intestinal obstruction	1
	Inguinal hernia	1
	Diverticulitis	1
Cardio-vascular	Total	14 (9)
	CHF exacerbation	6
	Chest pain	7
	PVD	1
Neurologic	Total	5 (3)
	CVA	4
	Confusion	1
Renal	Total	9 (6)
	Renal failure	6
	Hyperkalemia	1
	Nephrolithiasis	1
	Nephrectomy	1
Hematologic	Anemia/pancytopenia	5 (3)
Malignancy		7 (5)
Arthritis		1 (1)
Thyrotoxicosis		1 (1)
Hypoglycemia		1 (1)
Un-known diagnosis		8 (5)

COPD = Chronic obstructive pulmonary disease, CHF = Congestive heart failure, GI = Gastro-intestinal, PVD = Peripheral vascular disease, CVA = Cerebro-vascular accident. * Patients without a diagnosis are those who had not been discharged as yet or the discharge diagnosis was un-known.

## Data Availability

The data sets generated during this study are available from the corresponding author on request.
